# The effect of extraction of the lower first permanent molar on the developing third molar in children

**DOI:** 10.1177/14653125221093086

**Published:** 2022-04-27

**Authors:** Ian Murphy, Joseph Noar, Susan Parekh, Paul Ashley

**Affiliations:** 1 Department of Orthodontics, University College London (UCL) Eastman Dental Institute, London, UK; 2 Paediatric Dentistry, University College London (UCL) Eastman Dental Institute, London, UK

**Keywords:** occlusal development, aetiology of malocclusion and growth, minor oral surgery-orthodontic interface, interdisciplinary treatment, malocclusion

## Abstract

**Objective:**

To find the effect of extraction of the lower first permanent molar in children (aged 8–11 years) on the position and angle of the developing third molar.

**Design:**

Retrospective radiographic analysis.

**Participants:**

Two cohorts of participants were identified: an extraction group, who had extraction of one or more first permanent molars aged 8–11 years; and a non-extraction group, who retained all mandibular teeth. Both cohorts previously had panoramic radiographs taken at mean ages of 9.7 years (T1), before extraction, and 12.12 years (T2). In total, there were 61 third molars with an associated extracted first permanent molar and 60 third molars with an associated retained first permanent molar.

**Methods:**

A digital radiographic analysis was carried out on the panoramic radiographs to measure the movement of the third molar, vertically and horizontally, and its angle. The magnification of the T1 and T2 radiographs was calibrated. Reliability of the radiographic analysis was confirmed via intra- and inter-rater reliability tests. The extraction and non-extraction groups were compared via independent sample tests

**Results:**

The third molar moved significantly more mesial in the extraction group (*P* < 0.001) and the angle uprighted significantly more than the non-extraction group (*P* < 0.001). Vertically, the third molar moved inferiorly in both cohorts with no significant difference.

**Conclusion:**

In the developing dentition, extraction of the lower first permanent molar encouraged mesial movement and uprighting of the developing third molar. This may improve the likelihood of future eruption of the third molar.

## Introduction

The mandibular third permanent molar (M3) is highly variable in terms of its timing of formation, morphology and agenesis. The age range of calcification is normally 8–11 years (up to 15 years in extreme cases) and eruption is usually 17–21 years (Bishara and Andreasen, 1983; Garn et al., 1962). Impaction of mandibular third molars is common, with a worldwide incidence of 24.4% (Carter and Worthington, 2016), and third molar surgical molar removal has significant risks including bleeding, infection, alveolar osteitis and sensory disturbances of the inferior alveolar and lingual nerves in 0.35%–8.4% of cases (Sarikov and Juodzbalys, 2014). The M3 forms superficially in the ramus with its occlusal surface mesioangular. As more space is created from mandibular growth, it rotates into a more upright position (Richardson, 1978; Sicher and Dubrul, 1980) and submerges inferiorly as it moves from the ramus to the body of the mandible (Richardson, 1970). Third molars with an upright angle and space available are more likely to erupt (Richardson and Dent, 1974).

Poor prognosis first permanent molars (M1), often due to molar incisor hypo-mineralisation (MIH) or caries, may be extracted early to encourage mesial movement of the second permanent molar (Cobourne et al., 2014). The appropriately timed extraction of M1 may reduce the need for complex restorative treatment and subsequent later loss with resulting spacing. Early removal hopefully allows the developing second molar to erupt in a more mesial position, reduce the amount of residual spacing and the need for future orthodontic intervention. Generally, in the maxilla, the unerupted second permanent molar and M3 will produce a good occlusal position after extraction of the M1. In the mandible however, spacing and drifting often occurs (Cobourne et al., 2014; Sandler et al., 2000). Evidence has shown that if the mandibular M1 is extracted at 8–11 years, with the second permanent molar unerupted and the M3 present, spontaneous space closure via mesial migration of the second molar is significantly more likely to occur than if the second molar is erupted and/or the third molar is not present (Patel et al., 2016; Teo et al., 2016).

The panoramic radiograph is routinely used in orthodontics but has a degree of distortion and magnification (Catic et al., 1998; Leuzinger et al., 2010). The angle of individual teeth on the panoramic radiograph is different to the true angulation but the difference is rarely clinically significant (McKee et al., 2002). Although panoramic radiographs do not give a fully accurate representation, they do provide a high diagnostic yield and in the case of research, results should have a high enough level of significance and careful interpretation for the conclusion to be relied upon.

There is evidence that extraction of premolars followed by orthodontics improves the angle and space for the M3 (Livas and Delli, 2016); if the mandibular or maxillary second permanent molar is extracted before eruption of the M3, it is likely the M3 will erupt into an acceptable position (Huggins and McBride, 1978; Orton-Gibbs et al., 2001). Yavuz et al. (2006) conducted a review of patients who had asymmetric M1s extracted before the age of 12 years and were followed up to mid-adolescence. They found that the development and eruption of the M3 was significantly accelerated on the extraction side when compared to the control non-extraction side. However, there are no studies that have reviewed the effect of early extraction of the mandibular M1, at age 8–11 years, on the horizontal, vertical and angular change of the M3. Therefore, the aim of the present study was to investigate the effect of early extraction of first permanent molars on the developing M3 in the mandible, in terms of: (1) the anteroposterior (horizontal) movement; (2) the superior/inferior (vertical) movement; and (3) the angle of the long axis of the third molar.

## Materials and methods

The study design was a retrospective radiographic analysis.

Participants were identified from the paediatric theatre list from 1 April 2012 to 31 March 2019. Of the 9963 procedures recorded, records of children who had undergone extractions for poor prognosis M1s with a panoramic radiograph before extraction (T1) and at least one year after extraction (T2) were reviewed. The non-extraction group were also identified from the theatre list and consisted of patients who had panoramic radiographs for maxillary procedures. These procedures included exposure and bonding of ectopic maxillary teeth and extraction of maxillary teeth. These participants otherwise had normally developing mandibular teeth and were selected for panoramic radiographs at 8–11 years and again at least one year later, similar to the extraction group. Radiographs were taken using the PM 2002 EC Proline (Planmeca, Helsinki, Finland) and the staff followed standard operating procedures. Inclusion criteria included the following: M3 must be sufficiently developed with outline of occlusal surface present and panoramic radiographs taken at 8–11 years (T1) and at least 12 months later (T2). The T2 radiographs were taken as part of orthodontic assessment. Exclusion criteria included craniofacial syndromes, eruption anomalies, hypodontia, extraction of other permanent mandibular teeth, patients who have had orthodontics between T1 and T2, and poor-quality radiographs.

All the radiographs were plain film radiographs and were scanned at a resolution of 600 DPI by the principal investigator. A programme to analyse the radiographs was developed using the open access ‘ImageJ’ software by the medical physics department.

**Figure 1.**
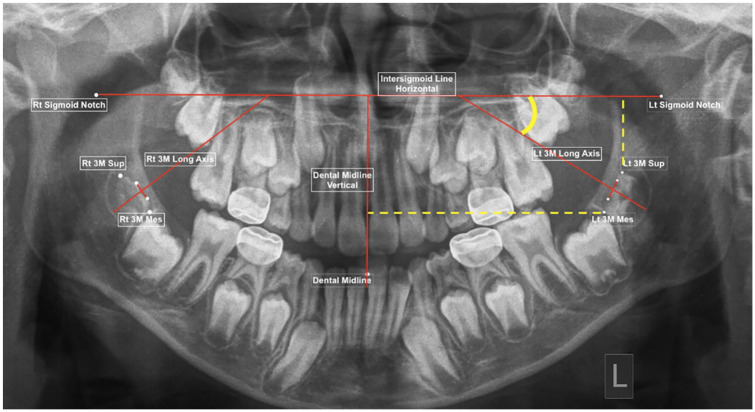
Panoramic radiograph landmarks and lines.

The panoramic radiograph landmarks, lines, linear and angular measurements were modified from previous studies reviewing the positional change of the mandibular M3 (Bayram et al., 2008; Miclotte et al., 2017; Tarazona et al., 2010) (Table 1, Figure 1). This established a horizontal reference, a line joining the left and right sigmoid notches, and the vertical reference, a line through the lower dental midline perpendicular to horizontal reference.

**Table 1.** Panoramic radiograph landmarks and line descriptions.

Landmarks and lines	
Rt3Mmes	Most mesial point of the developing right mandibular third molar
Rt3MSup	Most superior point of the developing right mandibular third molar
Rt Inf Sigmoid Notch	Most inferior point of the right sigmoid notch
Rt3MCusp tips	The two cusp tips of the developing right mandibular third molar
Mid	Contact point of lower dental midline
Lt3Mmes	Most mesial point of the developing left mandibular third molar
Lt3MSup	Most superior point of the developing left mandibular third molar
Lt Inf Sigmoid Notch	Most inferior point of the left sigmoid notch
Lt3MCusp tips	The two tips of the developing right mandibular third molar
Lines	
Inter-sigmoid (RtInfSig to LtInfSig)	Horizontal line joining most inferior point on right sigmoid notch to most inferior point on left sigmoid notch
Midline (mid perpendicular to inter-sig)	Perpendicular line drawn from contact point of lower dental midline to inter-sig line
Rt3MLongAx (perpendicular to line joining Rt3MCusp tips)	Perpendicular line through the midpoint of the line joining the mesial and distal cusp tips of the right developing third molar
Lt3MLongAx (perpendicular to line joining Lt3MCusp tips)	Perpendicular line through the midpoint of the line joining the mesial and distal cusp tips of the left developing third molar

Each radiograph required the identification of 17 radiographic landmarks. The software then created the lines and calculated linear measurements (in pixels) and angles (in degrees). No more than 10 radiographs were measured at a time before a 30-min break, to reduce fatigue.

### Outcomes

1. Horizontal movement of the M3 was assessed in reference to the vertical reference midline, the dental midline. The distance from the most mesial point of the developing M3 to the midline was recorded. The measurement is compared between the T1 and T2 in pixels.
2. The vertical movement of the developing M3 was measured as the distance from the most superior point of the M3 to the horizontal reference plane, the intersigmoid line, between T1 and T2 in pixels.
3. The angular change of the developing M3 was measured from the long axes of the M3 in relation to the intersigmoid line. The angle of the M3 is taken to be the outer angle formed by the longitudinal axis of the developing M3 to the horizontal reference line, recorded in degrees.

SPSS version 25 (IBM Corp., Armonk, NY, USA) was used for all data analysis.

### Magnification correction

The linear measurements were carried out in pixels because there is no ruler in a panoramic radiograph, as in a lateral cephalogram, to allow calibration to a millimetre scale. Although the radiographs were taken on the same machine with standard operating procedures, the magnification of a panoramic radiograph is not always the same for each radiograph. This is often due to very slight variation in the taking of the radiograph. This affects linear measurements, but angles are affected less so. In attempt to minimise this error and calibrate the two panoramic radiographs for the same individual, the mesio-distal widths of the mandibular second molars, left and right, and the lower central incisor were measured to create a calibration factor between T1 and T2. These teeth were selected as they represent the right, left and centre of the image.

### Reliability testing

Inter-rater calibration of radiographic measurements was carried out by the principal investigator and another author on 10 random panoramic radiographs. After a two-week wash-out period, the principal investigator again carried out the landmark identification to assess intra-rater calibration. Bland and Altman’s approach (Bland and Altman, 1986 ) and limits of agreement were calculated for each parameter.

### Sample size calculation

Sample size was calculated using the angulation outcome of a related study (Bayram et al., 2008), an agreed clinically significant difference of 15° in the M3 angulation, 90% power and a 5% level of significance. It was determined that 20 individuals would be required in each group, or 40 mandibular M3s in each group.

### Statistical methods

The three outcomes—horizontal and vertical position percentage change and angular change of the M3—were all assessed for normality by plotting the data on histograms. The differences in the three outcomes within the extraction and non-extraction groups were assessed by paired t-test or Wilcoxon signed-rank test, depending on normality, and between groups by independent sample *t*-test or Mann–Whitney *U*-test, depending on normality. Multiple linear regression was used to find the influence of sex, time between radiographs, ethnicity, if the left or right side was under investigation and whether the M1 was extracted.

## Results

### Participant flow

A total of 33 participants who had M1 extracted were identified. Of these, 28 had both mandibular M1s extracted with an associated developing M3. Two participants had both M1s extracted but one side had no associated M3; therefore, the side with no associated M3 was excluded. Three participants had one M1 extracted and the contralateral M1 was retained. For these three individuals, the extraction side was included in the extraction group and the non-extraction side in the non-extraction group.

A total of 29 individuals, who had neither mandibular first permanent molars extracted, were identified. Of these, one M1 had no associated M3.

There was a total of 61 M3s, with an extracted M1, included in the extraction group and a total of 60 M3s with a retained M1 in the non-extraction group.

Demographics and baseline data for the extraction and non-extraction groups are shown in Tables 2 and 3.

**Table 2.** Demographic data of participants.

Demographic data		n (%)
Total No. of participants		62
Extraction group		33 (53.22)
Non-extraction group		29 (46.78)
Sex – Extraction group	Female	20 (60.6)
	Male	13 (39.40)
Sex – Non-extraction group	Female	11 (37.93)
	Male	18 (62.07)
Ethnicity – Extraction group	Caucasian	23 (69.69)
	South Asian	5 (15.15)
	Afro-Caribbean	5 (15.15)
Ethnicity – Non-extraction group	Caucasian	18 (62.07)
	South Asian	2 (6.89)
	Afro-Caribbean	6 (20.69)
	Other	3 (10.34)

**Table 3.** Baseline data.

Intervals	Extraction group	Non-extraction group	*P* value
Age at T1 radiograph (years)	9.75 ± 0.96	9.64 ± 0.85	0.465
Age at extraction (years)	10.16 ± 1.2		
Age at T2 radiograph (years)	12.24 ± 1.52	11.97 ± 1.43	0.535
Time from radiograph T1 to T2	2.46 ± 1.21	2.29 ± 1.22	0.415
Time from radiograph T1 to extraction (months)	5.27 ± 3.21		
Time from extraction to radiograph T2 (years)	2.15 ± 1.05		
Angle of third molar to intersigmoid line (°) T1	34.38 ± 10.72	36.08 ± 11.82	0.411
Horizontal distance of third molar to midline (pixels) T1	1300.12 ± 332.24	1120.93 ± 593.23	0.868
Vertical distance of third molar to intersigmoid line (pixels) T1	405.37 ± 123.77	330.35 ± 175.18	0.097

Values are given as mean ± standard deviation.

The results of the calibration, inter-rater and intra-rater, were within a clinically acceptable range (Tables 4 and 5).

**Table 4.** Bland and Altman’s inter-rater reliability analysis.

	Bland and Altman’s approach (inter-rater)				
	Systematic error		Random error		
Parameter	MD	*P* value from paired *t*-test	SD of differences	Repeatability coefficient (BSRC)	Limits of agreement
Midline to Lt3Mmes l length (pixels)	–0.566	0.719	4.81	9.42	8.8612 to −9.9936
Midline to Rt3Mmes length (pixels)	–1.496	0.378	5.096	9.988	8.49 to −11.48
InterSig-Lt3MSup – length (pixels)	–0.514	0.874	9.926	19.454	18.94 to −19.969
InterSig-Rt3MSup –length (pixels)	–2.895	0.220	6.943	13.608	10.713 to −16.503
Intersig - Rt3MLongAx (°)	0.824	0.449	3.293	6.454	7.278 to −5.63
InterSig-Lt3MLongAx (°)	–2.198	0.471	4.1767	8.186	5.988 to −2.38

MD, mean difference; SD, standard deviation.

**Table 5.** Bland and Altman’s intra-rater reliability analysis.

	Bland and Altman’s approach (intra-rater)				
	Systematic error		Random error		
Parameter	MD	*P* value from paired t-test	SD of differences	Repeatability coefficient (BSRC)	Limits of agreement
Midline to Lt3Mmes length (pixels)	3.601	0.301	10.36	20.84	24.441 to −17.239
Midline to Rt3Mmes length (pixels)	–0.3620	0.896	8.525	16.709	16.347 to −17.071
InterSig-Lt3MSup – length (pixels)	0.2330	0.859	4.03	7.903	8.13621 to −7.67021
InterSig-Rt3MSup –length (pixels)	–0.1860	0.924	6.03	11.81	11.63096 to −12.003
Intersig - Rt3MLongAx (°)	–0.143	0.913	4.01	7.86	7.7182 to −8.004
InterSig-Lt3MLongAx (°)	0.0450	0.968	3.5	6.86	6.9085 to −6.8185

MD, mean difference; SD, standard deviation.

The difference between the horizontal, vertical and angle outcomes are summarised in Table 6. The M3 mesialised and the angle increased (tooth became upright) significantly more in the extraction group. There was no difference between T1 and T2 for the horizontal and angle of M3 outcome in the non-extraction group. In both groups, between T1 and T2, the M3 moved significantly inferiorly from the ramus to the body of the mandible but there was no difference between the groups.

**Table 6.** Comparison within groups at T1 and T2 and between the extraction and non-extraction groups.

Variable	T1	T2	Comparison of T1 and T2 within groups (*P*)	Change T1-T2	Comparison of change between groups (*P*)
Extraction group – horizontal distance of third molar to midline (pixels)	1300.12 ± 332.24	1171.38 ± 314.76	<0.001*	128.75 ± 135.39 (pixels)-9.48% ± 9.43%	<0.001^†^
Non-extraction group − horizontal distance of third molar to midline (pixels)	1120.93 ± 593.23	1104.58 ± 577.28	0.141*	16.35 ± 89.58 (pixels)-0.92% ± 7.08%	
Extraction group – vertical distance of third molar to intersigmoid line (pixels)	405.37 ± 123.77	504.11 ± 148.304	<0.001*	-98.73 ± 88.35 (pixels)26.51% ± 22.61%	0.067^†^
Non-extraction group – vertical distance of third molar to intersigmoid line (pixels)	330.35 ± 175.18	504.11 ± 148.3	<0.001*	-76.27 ± 67.69 (pixels)22.67% ± 14.01%	
Extraction group – angle of third molar to intersigmoid line (°)	34.35 ± 10.78	42.52 ± 11.36	<0.001^‡^ (95% CI = 5.49−10.84)	8.17 ± 10.45 (°)	<0.001^§^ (95% CI = 4.16–11.17)
Non-extraction group – angle of third molar to intersigmoid line (°)	36.08 ± 11.81	36.58 ± 10.82	0.665^‡^ (95% CI = −2.81 to 1.8)	0.5 ± 8.93 (°)	

* Comparison of T1 and T2 measurements, Wilcoxon signed-rank test.

† Comparison between groups, Mann–Whitney *U*-test.

‡ Comparison of T1 and T2 measurements, paired *t*-test.

§ Comparison between groups, independent samples *t*-test.

CI, confidence interval.

The multiple linear regression (Table 7) showed if sex, time between radiographs, ethnicity or side had a significant effect on the angular or horizontal change. The time between the panoramic radiograph was a significant factor for the vertical change, i.e. the greater the time between the panoramic radiographs the more inferior the M3 moved in a vertical sense, from the ramus to the body of the mandible.

**Table 7.** Multiple linear regression.

M3 parameter	Model	*P* value
Horizontal change	M1 extraction	<0.001
	Sex	0.135
	Time between radiographs	0.116
	Left or right	0.308
	Ethnicity	0.111
Vertical change	M1 extraction	0.434
	Sex	0.608
	Time between radiographs	0.006
	Left or right	0.589
	Ethnicity	0.087
Angulation change	M1 extraction	<0.001
	Sex	0.803
	Time between radiographs	0.08
	Left or right	0.110
	Ethnicity	0.411

## Discussion

**Figure 2.**
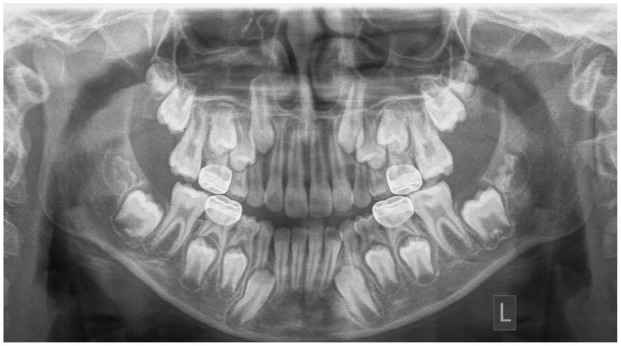
T1 pre-extraction example.

**Figure 3.**
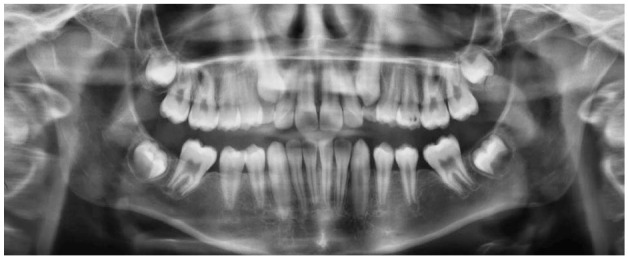
T2 post-extraction example.

**Figure 4.**
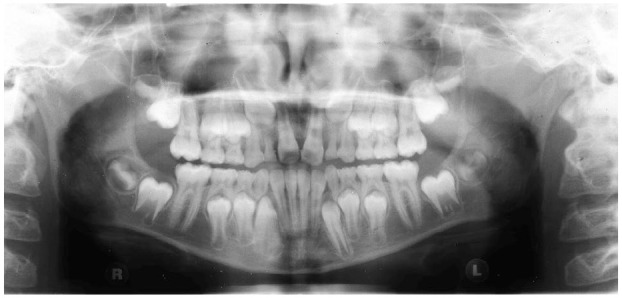
T1 non-extraction example.

**Figure 5.**
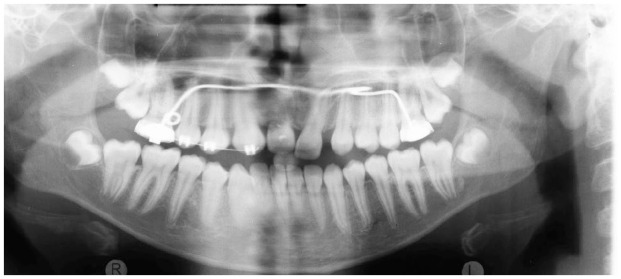
T2 non-extraction example.

The results of this investigation showed that extraction of the M1 in the mandible during the developing dentition, at 8–11 years, has a mesialising and uprighting effect on the developing M3. This change in the position of the M3 may improve future eruption and prevent pathology associated with impaction. This movement is illustrated in examples of extraction and non-extraction cases (Figures 2–5). In the extraction group, the M3 moves more mesially and uprights.

Despite the positive results found in this study, there were limitations.

Due to the retrospective nature of the study, it was not possible to include a physical assessment of the crowding or skeletal pattern, which could have given us more information of the overall dental development. Although the multiple linear regression found no difference between the groups, they were not perfectly matched. The proportion of boys and girls and ethnicities were different; if the study had been prospective this could have been rectified. To strengthen this study a prospective approach could have been used; however, even with that design it may have been difficult to justify further radiation for research purposes alone. A further improvement in this study would have been to have followed up the patients until eruption of the M3. The current study may justify the expense in carrying out such a longitudinal observational investigation. Randomised controlled trials (RCTs) in this field have not been done, most likely because of difficulty in recruitment, ethics and length of follow-up. A previous attempted RCT was terminated due to poor recruitment (Bearn, 2012).

It is recognised that panoramic radiographs have limited established radiographic analyses and can suffer from varied magnification. However, they are routinely used to assess the developing dentition and are therefore easily justified during orthodontic assessment. To prevent error, we have chosen to be conservative at the level of significance to ensure that any significant changes are real. Measuring a linear distance on a tomograph, a two-dimensional representation of a three-dimensional object, is challenging. As there is no ruler on panoramic radiographs, it was decided to use pixels to measure linear distances and apply a magnification calibration between radiographs, T1 and T2, so they were comparable. Although the magnification calibration in this study was not perfect, including a control for magnification did attempt to mitigate this and the statistical analysis showed that the analysis used in this study was reliable.

The results showed that the horizontal distance of the M3 to midline reduced by 9.48% in the extraction group and by 0.92% in the non-extraction group and was statistically significant. A 9.48% difference in the mesialising of the M3 after extraction of the M1 may not be clinically significant in isolation; however, it does show the extraction has a mesialising effect in the early stages of M3 development. This may continue during development.

For the vertical change of the M3, both extraction and non-extraction groups displayed a significant movement in an inferior direction. This is to be expected from an early developing M3; it moves from the ramus to the body of the mandible in its early stages (Richardson, 1970).

For the angle outcome, the angle of the M3 in the extraction group increased by 8.17° compared to 0.5° in the non-extraction group. This may not be clinically significant at this stage; the sample size calculation was based on a 15° clinically significant difference, but if there is a significant angulation change in a short period of observation then this may continue to upright. This supports the evidence that when the space is present, M3s tend to upright and are more likely to erupt without complication (Richardson and Dent, 1974; Richardson, 1975). A larger sample in a prospective study may find a greater difference between groups with time.

From these results, it could be said that when a poor prognosis lower first permanent molar is removed in a child, there is a less likely chance of the third molar becoming impacted in the future. This alone could not justify the first permanent molar extraction but could be a factor in the treatment planning, i.e. extraction of the poor prognosis first permanent molar could remove the long-term restorative burden of the tooth as well prevent potential future pathology associated with third molars and their removal.

## Conclusion

In this study, extraction of the M1 in the mandible during the developing dentition, at 8–11 years, had a significant mesialising and uprighting effect on the developing M3. This may improve future eruption and prevent pathology associated with impaction.
